# A TrxR inhibiting gold(I) NHC complex induces apoptosis through ASK1-p38-MAPK signaling in pancreatic cancer cells

**DOI:** 10.1186/1476-4598-13-221

**Published:** 2014-09-25

**Authors:** Xinlai Cheng, Palvo Holenya, Suzan Can, Hamed Alborzinia, Riccardo Rubbiani, Ingo Ott, Stefan Wölfl

**Affiliations:** Institut für Pharmazie und Molekulare Biotechnologie, Ruprecht-Karls-Universität Heidelberg, Im Neuenheimer Feld 364, 69120 Heidelberg, Germany; Institute of Medicinal and Pharmaceutical Chemistry, Technische Universität Braunschweig, Beethovenstrasse 55, 38106 Braunschweig, Germany

**Keywords:** Gold(I) NHC complex, Apoptosis, Thiolredoxin Reductase inhibitor, ASK1, p38-MAPK, Anti-cancer drug, ROS, PDAC

## Abstract

**Background:**

Cancer cells in the advanced stage show aberrant antioxidant capacity to detoxify excessive ROS resulting in the compensation for intrinsic oxidative stress and therapeutic resistance. PDAC is one of the most lethal cancers and often associated with a high accumulation of ROS. Recent studies identified gold(I) NHC complexes as potent TrxR inhibitors suppressing cell growth in a wide spectrum of human malignant cell lines at the low micromolar concentration. However, the mechanism of action is not completely elucidated yet.

**Methods:**

To understand the biological function of gold(I) NHC complexes in PDAC, we used a recently published gold(I) NHC complex, MC3, and evaluated its anti-proliferative effect in four PDAC cell lines, determined by MTT and SRB assays. In further detailed analysis, we analyzed cellular ROS levels using the ROS indicator DHE and mitochondrial membrane potential indicated by the dye JC-1 in Panc1. We also analyzed cell cycle arrest and apoptosis by FACS. To elucidate the role of specific cell signaling pathways in MC3-induced cell death, co-incubation with ROS scavengers, a p38-MAPK inhibitor and siRNA mediated depletion of ASK1 were performed, and results were analyzed by immunoblotting, ELISA-microarrays, qRT-PCR and immunoprecipitation.

**Results:**

Our data demonstrate that MC3 efficiently suppressed cell growth, and induced cell cycle arrest and apoptosis in pancreatic cancer cells, in particular in the gemcitabine-resistant cancer cells Panc1 and ASPC1. Treatment with MC3 resulted in a substantial alteration of the cellular redox homeostasis leading to increased ROS levels and a decrease in the mitochondrial membrane potential. ROS scavengers suppressed ROS formation and rescued cells from damage. On the molecular level, MC3 blocked the interaction of Trx with ASK1 and subsequently activated p38-associated signaling. Furthermore, inhibition of this pathway by using ASK1 siRNA or a p38 inhibitor clearly attenuated the effect of MC3 on cell proliferation in Panc1 and ASPC1.

**Conclusions:**

Our results confirm that MC3 is a TrxR inhibitor and show MC3 induced apoptosis in gemcitabine-resistant PDACs. MC3 mediated cell death could be blocked by using anti-oxidants, ASK1 siRNA or p38 inhibitor suggesting that the Trx-ASK1-p38 signal cascade played an important role in gold(I) NHC complexes-mediated cellular damage.

**Electronic supplementary material:**

The online version of this article (doi:10.1186/1476-4598-13-221) contains supplementary material, which is available to authorized users.

## Background

The discovery of *cis*-diamminedichloroplatinum (cisplatin) as an antitumor agent by Rosenberg in 1965 was a hallmark in inorganic medicinal chemistry [1]. Although cisplatin as well as its derivatives, carboplatin and oxaliplatin, are correlated with high toxicity, limited selectivity and a high ratio of drug resistance [2, 3], they still are widely used as effective chemotherapeutic substances [4, 5]. In the last three decades several other metal-based compounds were synthesized with the expectation to overcome therapeutic limitations, which include ruthenium- [6, 7], rhodium- [8], iridium- [8] and gold-complexes [9, 10]. While cisplatin and its derivatives exert their anti-proliferative activity through DNA damage [11], and a specific cellular cytotoxic response [12], organo-metal complexes can also act through other mechanisms [13]. For gold-complexes a strong inhibition of thiol-containing enzymes like Thioredoxin Reductase (TrxR) has been demonstrated due to the high native affinity of gold to thiol-group [9, 10].

The rapid proliferation of cancer cells requires high metabolic activity, which includes increased glycolysis but also an elevation of other metabolic reactions. Due to this increase in metabolic rate, cancer cells, in particular, those in advanced stage are prone to high oxidative stress caused by abundant reactive oxygen species, considered to mainly originate from electronic leakage of mitochondrial respiratory complexes [14, 15]. Interestingly, a moderated increase in ROS level in cancer cells is an indicator of DNA damage, genomic instability, proliferation, migration and formation of metastasis, while cells with an excessive accumulation of ROS will typically undergo irreversible cell death [16, 17]. There are strong evidences that adaptive mechanisms enable cancer cells to escape from oxidative damage [18, 19] by means of over-expressing ROS scavengers including Thioredoxin (Trx) and/or Glutathione (Glu) and pro-survival proteins like Bcl-xl [20]. Activation of both, redox control and anti-apoptotic signaling will help cancer cells to cope with lethality in response to aberrant ROS levels.

Trx and TrxR provide a coupled redox system, which is required for redox reactions in biosynthetic pathways and is involved in the control of redox homeostasis in cells [19, 21]. Trx, a reduction/oxidation protein, can be oxidized, e.g. by abundant ROS, which leads to formation of a disulfide bridge. The reduction by TrxR re-activates Trx providing a circuit for sequential turnover in multiple oxidation/reduction cycles [19, 21]. In its reduced form Trx inhibits apoptosis signal-regulating kinase 1 (ASK1) and the downstream mitogen-activated protein kinase p38 (p38-MAPK). Upon accumulation of ROS, Trx is oxidized and ASK1 is activated leading to apoptotic cell death [22, 23]. In several cancer cells, over-expression of Trx increases the capacity for ROS, which leads to increased drug resistance and promotes tumor progression [24]. Therefore, several small molecules targeting the Trx-TrxR system have been developed to preferentially induce cell death in malignant cells [25–27] due to the increased dependence of these cells on the anti-oxidative activity of the Trx-TrxR system [28–30].

Recently, we presented several gold(I) *N*-heterocyclic carbene (NHC) complexes as potent inhibitors of TrxR, showing clear and strong anti-proliferative effects on a broad spectrum of tumor cells [9, 10]. In this article, we performed a more detailed analysis of the underlying molecular mechanism leading to cell death and found that [di-(1,3-diethylbenzylimidazol-2-ylidene)]gold(I) iodide (called MC3), a TrxR inhibitor, showed a high anti-proliferative effect on pancreatic ductal adenocarcinoma (PDAC). This effect was clearly associated with increased ROS production and activation of p38-MAPK. ROS scavengers, inhibition of p38 signaling with a specific inhibitor or depletion of ASK1 attenuated MC3-induced apoptosis. In addition, we could clearly show that treatment with MC3 interrupted binding of Trx to ASK1. Our findings provide a detailed mechanism for the activation of ASK1-p38 signaling following interference with the Trx-TrxR redox balance as a major mechanism in gold(I) compounds-mediated cell death.

## Results

### Anti-proliferative effect of MC3 in gemcitabine-resistant pancreatic cancer cells

**Figure 1:**
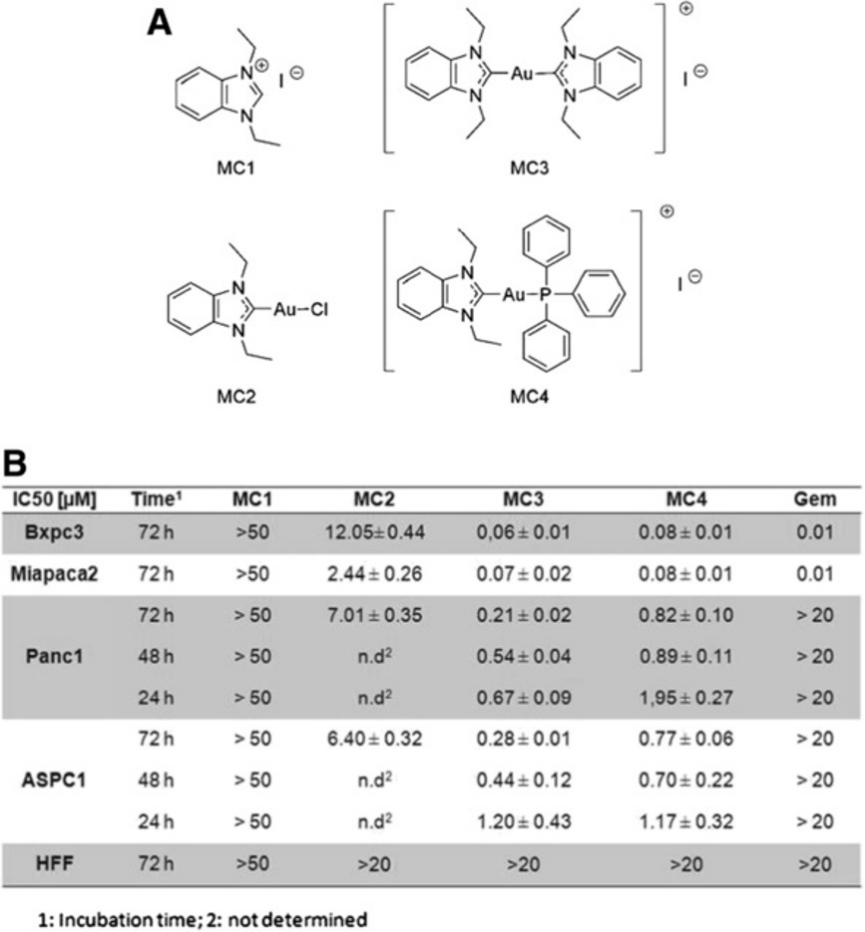
**IC**_**50**_ **values of gold(I) NHC complexes. (A)** Structures of compounds. **(B)** IC_50_ values of compounds in Bxpc3, Miapaca2, Panc1 and ASPC1, as well as HFF cells, at different times as indicated. The detailed procedure is described in the experimental section. The IC_50_ [μM] values were calculated from dose–response curves in three independent experiments.

Gold(I) NHC complexes had been identified as potent anti-tumor agents with IC50 value in the low micromolar concentration range in various cancer cell lines, which represent different tumors including MCF7 (breast cancer), HT29 and HCT116 (colon cancer), and HepG2 (liver cancer) [9, 10]. Here we investigated their effects on pancreatic cancer cells, one of the most difficult to treat cancers with only 5% of 5-year survival in patients [31]. In our first analysis, we evaluated the cytotoxicity of the gold(I) metal-complexes MC2, MC3 and MC4 in four pancreatic cancer cell lines, namely Bxpc3, Miapaca2, Panc1 and ASPC1 using MTT assay. The free ligand (MC1), 1,3-diethylbenz imidazolium iodide, and the well-known chemotherapeutic drug gemcitabine (Gem) were included as references [32]. The structures were listed in Figure 1A. After 72 h treatment we observed that Gem was sufficient to block 50% of cell growth in Bxpc3 and Miapaca2 cells at 10 nM, whereas IC50 values were more than 20 μM in Panc1 and ASPC1 (Figure 1B). In MC series, we could not detect any anti-proliferative effect of the organic ligand (MC1) at 50 μM in all of the four cell lines. By contrast, MC2, possessing a chloride as leaving group, exhibited a good inhibitory effect with IC50 values of 12.05 ± 0.44, 2.44 ± 0.26, 7.01 ± 0.35 and 6.40 ± 0.32 μM in Bxpc3, Miapaca2, Panc1 and ASPC1 respectively (Figure 1B), while MC4, carrying triphenylphosphine instead of chloride, inhibited cell growth with IC50 values of 0.08 ± 0.01, 0.08 ± 0.01, 0.82 ± 0.01 and 0.77 ± 0.06 μM in the respective cell lines (Figure 1B). Interestingly, the most potent compound was MC3, substituted with a second benzimidazolylidene moiety, whose IC50 values were 0.06 ± 0.01, 0.07 ± 0.02, 0.21 ± 0.02 and 0.28 ± 0.01 μM in corresponding PDACs (Figure 1B). Additionally, we also measured the short-term effect of MC3 and MC4 and detected remarkable toxicity of MC3 and MC4 after 24 h and 48 h treatment in Panc1 and ASPC1 (Figure 1B). Of note, MC3, MC4 and Gem exhibited low toxic effects on human neonatal foreskin fibroblasts (HFF), implicating they might preferentially kill cancer cells (Figure 1B). The MTT assay is a colorimetric proliferation and cell viability assay, which is based on the measurement of the activity of mitochondrial succinate dehydrogenase [33]. Since mitochondria had been shown to be a direct target of gold(I) NHC complexes [9, 10], we also used total protein quantification with sulforhodamine B, known as SRB assay, to measure cellular growth and proliferation [34]. The results obtained were similar in both assays. Since Panc1 and ASPC1 are resistant to Gem, we focused on these two cell lines, in particular Panc1, which possesses mutations in p53, in our further experiments [35, 36].

### Cell cycle arrest, reduction of ROS formation and induction of apoptosis upon MC3 treatment

**Figure 2:**
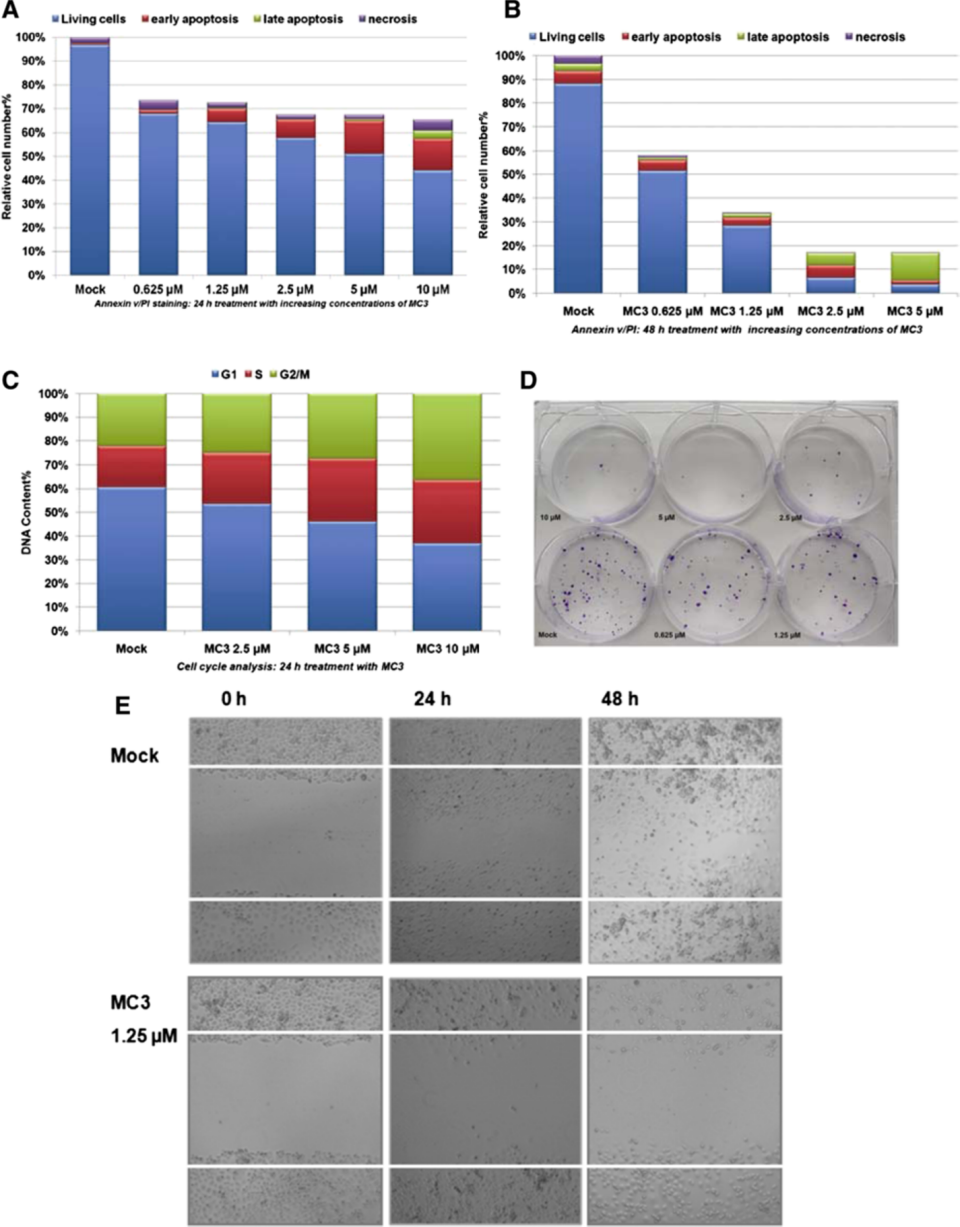
**MC3 induced cell apoptosis in Panc1.** The cells were incubated with various concentrations of MC3 for 24 h **(A)** and 48 h **(B)**, and analyzed by FACS using annexin v/PI staining. **(C)** S and G2/M phase cell arrests occurred 24 h after treatment in Panc1. The relative DNA content was analyzed by FACS using PI staining. The percentage of cells was determined by using trypan blue assay. **(D)** MC3 prevented colony formation in Panc1. Panc1 cells were treated with increasing concentrations of MC3 for 24 h, re-plated at 2000 cells/well in a new 6-well plate and detected with crystal violet after 3 weeks cultivation. **(E)** MC3 inhibited cell migration in wound healing assay. Panc1 cells were incubated with DMF as mock or MC3 (1.25 μM) under optimized condition as described in experimental section, and photos were taken at 0 h, 24 h and 48 h.

To shed light on the mechanism of cell death we stained treated cells with FITC-conjugated annexin v, which binds to phosphatidylserine translocated to the outer cell membrane layer during apoptosis [37, 38], and propidium iodide (PI), which is only taken up after membrane damage, to distinguish necrotic cells. Panc1 cells were incubated with increasing concentrations of MC3 for 24 and 48 hrs. After treatment cells were counted and analyzed by FACS (fluorescence activated cell sorting). Treatment not only reduced cell number, but also led to a concentration-dependent increase in the population of annexin v positive cells compared to DMF treatment as mock already after 24 h (Figure 2A). The significance of apoptotic cell death is even more clearly visible after 48 h treatment. Cell numbers are further reduced and the relative amount of early apoptotic and late apoptotic cells is increased in the remaining cell population (Figure 2B). Analysis of cell cycle distribution in intact cells after 24 h treatment, detected accumulation of cells at S and G2/M phases (Figure 2C), indicating an accompanying cell cycle arrest. To investigate the ability of cells to grow into a colony after treatment, we performed a colony formation assay by seeding equal amount of cells, pre-incubated with increasing concentrations of MC3 for 24 h. After 3 weeks of continuous cultivation, cells were stained with crystal violet. Hardly any visible colony could be found, when cells were treated with 5 μM or 10 μM during pre-incubation (Figure 2D). We also performed a wound healing assay, under conditions optimized for low cell proliferation (0.5% serum) with an assay-specific optimized concentration of MC3 (1.25 μM) to minimize toxicity. After 48 h incubation in this condition, we hardly saw any cell able to enter the gap generated with a scratch in the cell layer, indicating reduced cell mobility in presence of MC3 (Figure 2E).

**Figure 3:**
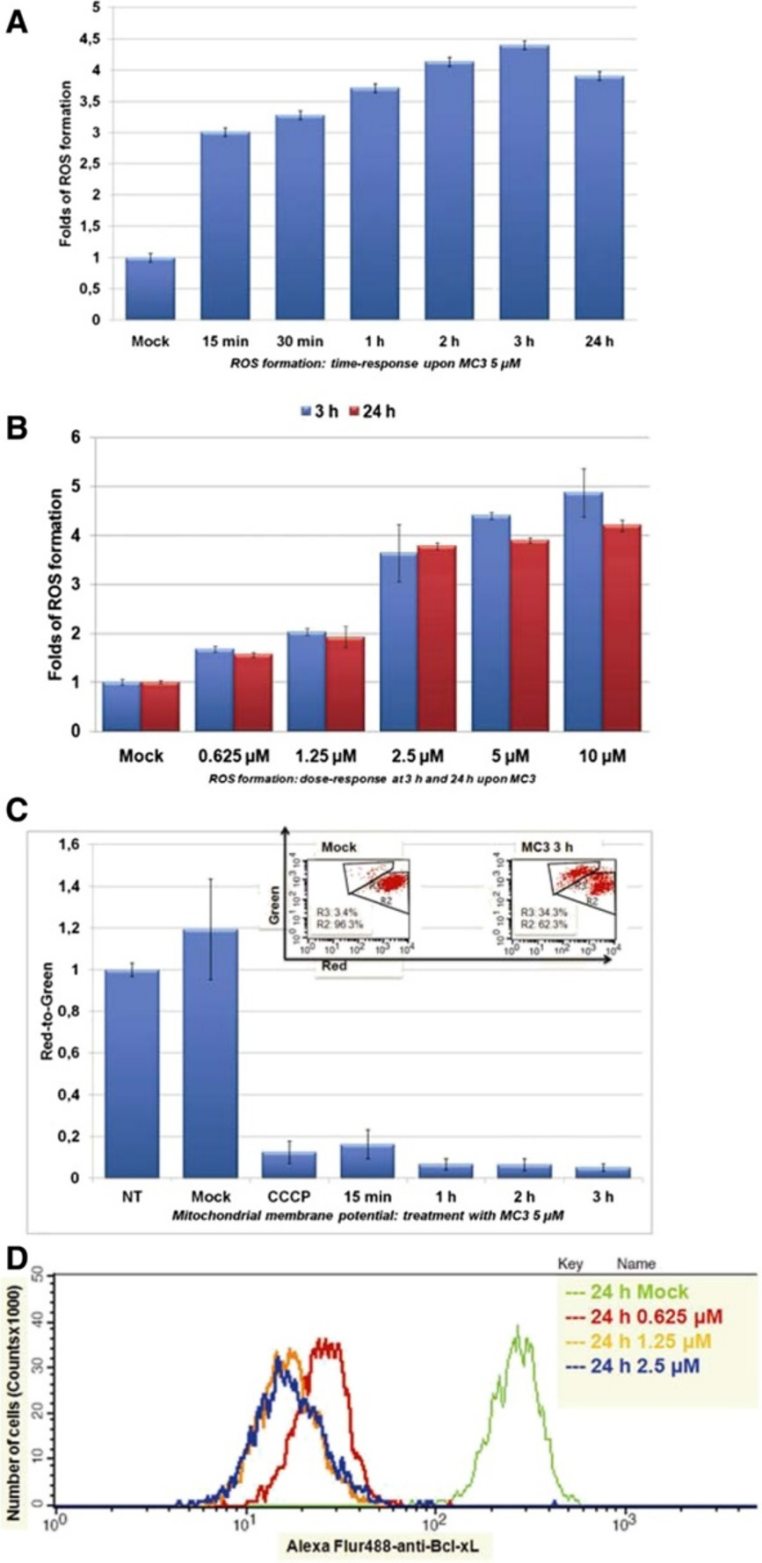
**MC3 interfered cellular redox homeostasis.** MC3 promoted ROS formation in time- **(A)** and concentration **(B)**-dependent manner. Panc1 cells were incubated with MC3 as indicated and ROS formation was measured using DHE. Data were normalized to the value of DMF-treated cells. **(C)** MC3 reduced membrane potential. Panc1 cells were treated with MC3 5 μM at indicated time points and collected for the measurement as described in the experimental section. The data show a clear reduction in the ratio of red population defined as R2 to green population defined as R3. DMF was used as mock and CCCP (carbonyl cyanide 3-chlorophenylhydrazone) as the positive control. Data were normalized to the value of non-treated cells. The raw data of mock and MC3 treatment for 3 h are depicted in an inset picture. **(D)** MC3 inhibited Bcl-xL activity. The amount of Bcl-xL was determined by FACS using Alexa Flur® 488-conjugated Bcl-xL antibody in 24 h MC3-treated Panc1 cells. DMF was used as mock.

Since 5 μM of MC3 was sufficient to obtain a significant amount of apoptotic cells after 24 h treatment (Figure 2A), we decided to assess the formation of ROS in a time-dependent study at this concentration. We used dihydroethidum (DHE) and FACS analysis for ROS detection. ROS levels were clearly changed, showing a dramatic elevation as early as 15 min (3-fold over mock) after treatment started, with a maximum after 3 h (4.4-fold) and 3.9-fold (over mock) after 24 h (Figure 3A). The slight decrease at the later time point might be explained by an increased compensation in cancer cells attempting to overcome MC3-indcued excessive ROS for survival. We further analyzed ROS formation at various concentrations at 3 h and 24 h, both showing a concentration-dependent increase (Figure 3B) in good agreement with previous findings that MC3 acts as a potent TrxR inhibitor [9, 10]. Since increase of ROS may be initiated from dysfunctional mitochondria [39], we analyzed the mitochondrial membrane potential, with the cytofluorimetric, lipophilic cationic dye, 5,5′,6,6′-tetrachloro-1,1′,3,3′- tetraethylbenzimidazolyl-carbocyanine iodide (known as JC-1) in Panc1 upon MC3 treatment [40]. We found a constant decrease in the ratio of red to green fluorescence indicating a decline in the mitochondrial membrane potential in MC3-treated cells (Figure 3C). In addition, Bcl-xl, an anti-apoptotic protein, was concentration-dependently reduced after 24 h (Figure 3D). All above observations together provide a clear indication that MC3 is a small molecule efficiently inhibiting cell proliferation by targeting redox regulation in Panc-1 cells.

### Activation of the ASK1-p38-MAPK pathway in response to MC3

**Figure 4:**
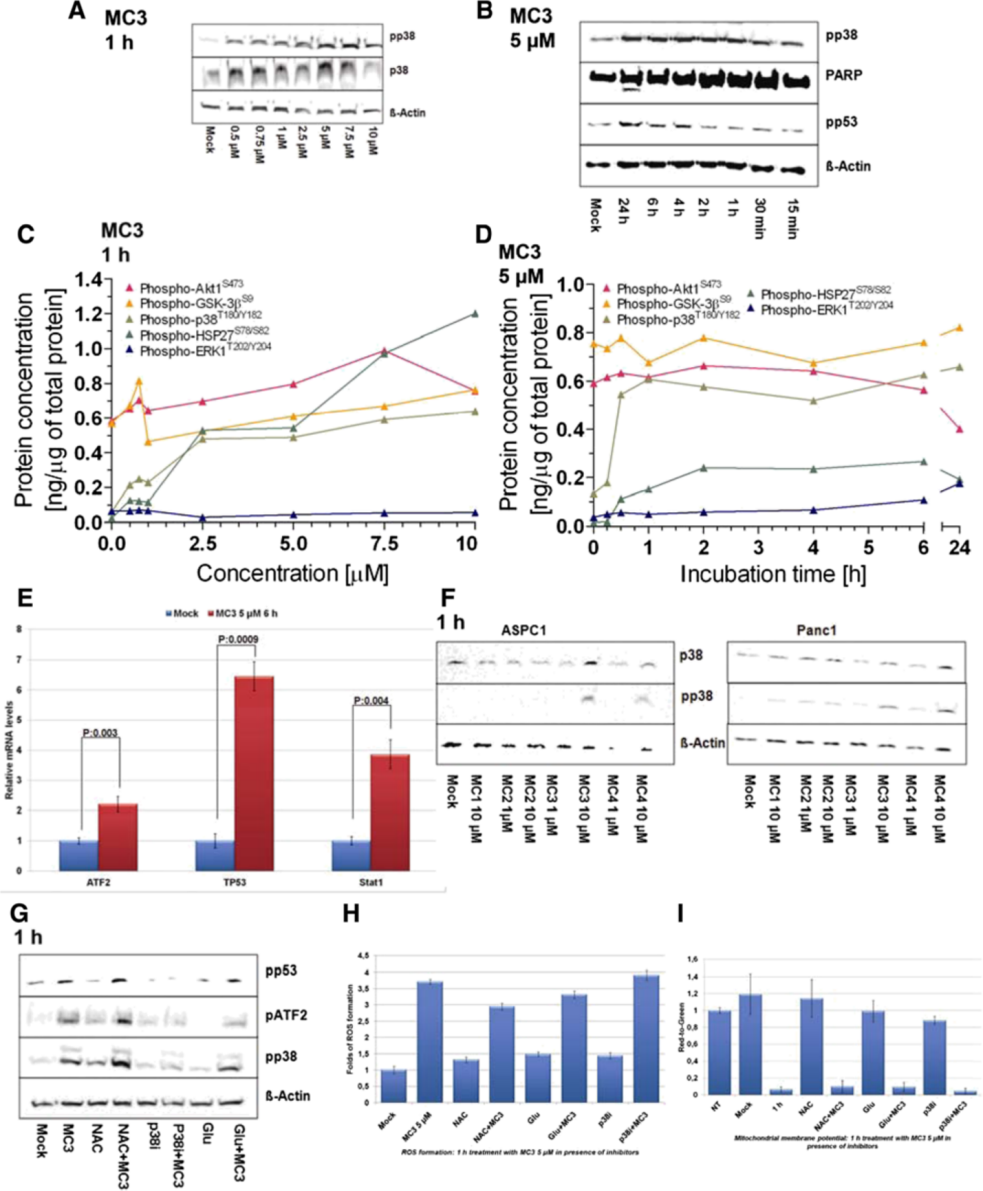
**Role of P38-MAPK cascade in MC3-mediated cellular apoptosis. (A)** MC3 activated p38-MAPK. Panc1 cells were incubated with increasing concentrations of MC3 as indicated for 1 h. Whole cell lysates were subjected to immunoblot and probed with p38-MAPK and phospho-p38-MAPK antibodies. DMF was used as mock, ß-Actin as loading control and 40 μg proteins were loaded per lane. **(B)** MC3 activated p53 and induced PARP cleavage. Panc1 cells were treated with 5 μM MC3 as shown. Phospho-p53 and PARP cleavage were detected with respective antibodies in immunoblot. Phosphorylation of p38-MAPK, HSP27, Erk1/2, GSK3ß and Akt were determined by microarray analysis in concentration- **(C)** and time- **(D)** dependent studies. **(E)** MC3 activated expressions of p38-MAPK-related genes. Panc1 cells were incubated with MC3 5 μM for 6 h and the expression of ATF2, TP53 and Stat1 were analyzed by qRT-PCR. Data were normalized to the value of DMF-treated cells, shown is the mean ± SD of quadruplicates and representing three independent experiments. **(F)** p38-MAPK was activated by MC3 and MC4 treatment in ASPC1 and Panc1 cells. The cells were treated with gold(I) NHC complexes or ligand for 1 h. Phospho- and total-p38-MAPK were detected with corresponding antibodies. **(G)** p38 inhibitor (p38i), but not ROS scavengers, blocked MC3-activated p38-MAPK pathway within 1 h incubation. Panc1 cells were incubated with 5 μM MC3, 10 mM NAC, 10 μM p38i, 5 mM Glu or in combination as indicated for 1 h. Phosphorylation of p38-MAPK, ATF2 and p53 were detected by using specific antibodies. ROS scavenger did not influence on MC3-caused ROS formation **(H)** or mitochondrial membrane potential alternation **(I)**. *The densitometric analyses of corresponding immunoblots are provided in supporting information.*

Reduced Trx can bind to ASK1 and consequently blocks ASK1 activity [22]. Therefore, we postulated that inhibition of TrxR by MC3 might keep Trx in the oxidized state, which could release free ASK1 and in turn activate the p38-MAPK pathway to promote cell apoptosis. Using immunoblotting we clearly detected concentration-dependent activation of phospho-p38-MAPK (T180/Y182) upon MC3 for 1 h (Figure 4A and Additional file 1: Figure S1). We found a fast activation of p38-MAPK by MC3 (<30 min), which persisted over the test period of 24 h (Figure 4B and Additional file 1: Figure S2). We also observed phosphorylation of p53 and PARP cleavage after 24 h (Figure 4B and Additional file 1: Figure S2), hallmarks of cell apoptosis, in line with our above findings that MC3 induced cell cycle arrest and apoptosis. Analysis of phosphorylated p38-MAPK and HSP27 using quantitative phosphoprotein ELISA microarrays [41] confirmed time- (Figure 4C) and concentration-dependent (Figure 4D) activation of p38-MAPK signaling upon MC3 [42], while phosphorylation of other signal transduction kinases like Erk1/2, Akt and GSK3ß was not significantly changed.

To further understand the profound role of the ASK1-p38-MAPK cascade in the cellular response to MC3, we isolated RNA after treatment with MC3 (5 μM) for 6 h and analyzed expression of p38-MAPK downstream genes including ATF2 [43], Stat1 [44] and TP53 [45]. Expression of ATF2, Stat1 and TP53 was clearly induced upon MC3 treatment (Figure 4E), confirming the activation of p38-MAPK signaling by MC3 in Panc1 cells. We further tested if gold(I) NHC complexes could generally activate p38-MAPK in Panc1 and ASPC1 cells and found abundant phospho-p38-MAPK only in presence of MC3 or MC4 (Figure 4F and Additional file 1: Figure S3), implicating a relationship between the anti-proliferative effect of gold(I) NHC complexes and p38-MAPK activity.

### p38 and ROS are involved in MC3-induced apoptosis

To confirm this mechanism on the molecular level, we next used a chemical p38-MAPK inhibitor (p38i, SB203580) in a co-treatment with 5 μM MC3 in Panc1 for 1 h. The results obtained confirmed that this combination could not only block MC3-induced activation of p38-MAPK-associated proteins, such as p38-MAPK and ATF2, but also activation of p53 (Figure 4G and Additional file 1: Figure S4), suggesting that p38-MAPK signaling was directly involved in MC3-mediated cell death. Since MC3 is a TrxR inhibitor, we next investigated if adding a ROS scavenger could prohibit activation of the p38-MAPK cascade. Two anti-oxidants, N-actetyl-L-cysteine (NAC, 10 mM) and reduced glutathione (Glu, 5 mM), were used in co-incubation with MC3 (5 μM) for 1 h. Surprisingly, both antioxidants could not alter the level of phospho-p38-MAPK and -p53 (Figure 4G and Additional file 1: Figure S4). Importantly, the higher level of ROS (Figure 4H) and the lower mitochondrial membrane potential (Figure 4I) compared to DMF control could still be detected at this early time point (1 h) of MC3 treatment in presence of NAC and Glu.

**Figure 5:**
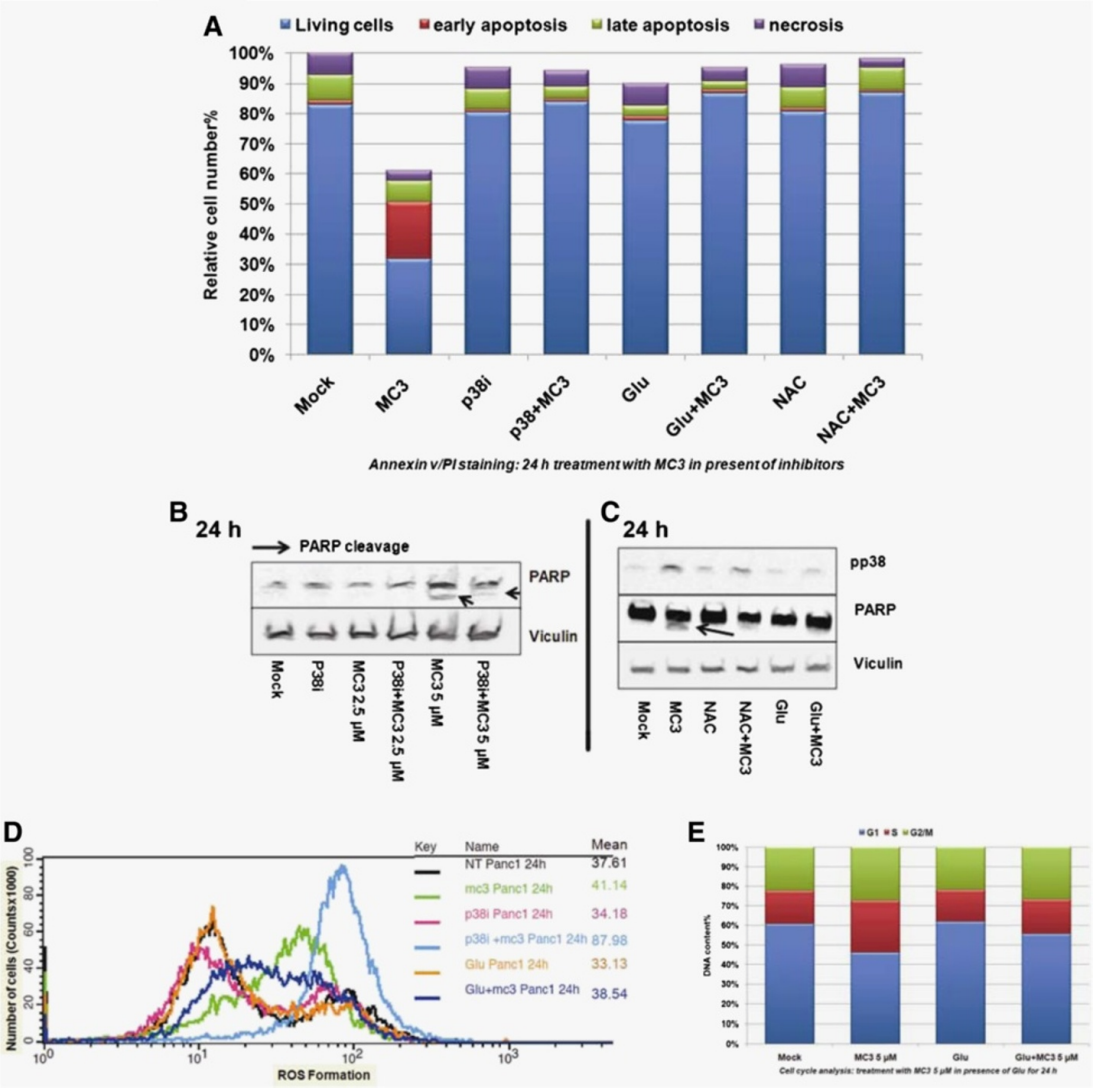
**Opposite effects of ROS scavenger and p38i against MC3. (A)** ROS scavengers and p38i rescued MC3-induced cell apoptosis in 24 h treatment. Panc1 cells were incubated with 5 μM MC3, 10 μM p38i, 10 mM NAC or 5 mM Glu or in combination as indicated. The cell apoptosis was analyzed by using annexin v/PI staining. Immunoblots showing: **(B)** p38i prevented PARP cleavage caused by MC3; and **(C)** reduced phospho-p38 levels and inhibition of PARP cleavage after co-treatment with ROS scavengers. Panc1 cells were incubated with inhibitors or MC3 or in combination for 24 h as indicated. Vinculin was used as loading control. **(D)** Alternation of ROS production in combination of MC3 with inhibitors. ROS formation was analyzed by FACS in Panc1 cells treated with various compounds or compounds combination for 24 h. The mean values were listed. **(E)** Glu compensated MC3-mediated cell cycle arrest after 24 h treatment. *The densitometric analyses of corresponding immunoblots are provided in supporting information.*

We further questioned if the p38 inhibitor (p38i) and anti-oxidant treatment are able to prevent cells from MC3-induced damage. Thus, we exposed Panc1 to MC3 in presence of ROS scavengers or p38i for 24 h and analyzed cell populations labeled with annexin v/PI as described before. The results clearly showed that ROS scavengers and p38i significantly counteracted the toxicity of MC3 (Figure 5A). Further immunoblotting data demonstrated that PARP cleavage induced by MC3 disappeared in co-treatment with p38i or ROS scavengers after 24 h (Figure 5B, Figure 5C and Additional file 1: Figure S5). We also, found that ROS scavengers considerably reduced the activity of p38-MAPK (Figure 5C and Additional file 1: Figure S5), as well as ROS accumulation (Figure 5D) and cell cycle arrest (Figure 5E) after 24 h, indicating a compensatory effect of ROS scavenging against MC3 in a long-term treatment. Interestingly, p38i attenuated cellular apoptosis caused by MC3, but enhanced ROS level (Figure 5D), suggesting that cells under severe oxidative stress might employ p38-MAPK to cope with excessive ROS.

### MC3 disrupts ASK1-Trx interaction and ASK1-deficient cells are resistant to MC3

**Figure 6:**
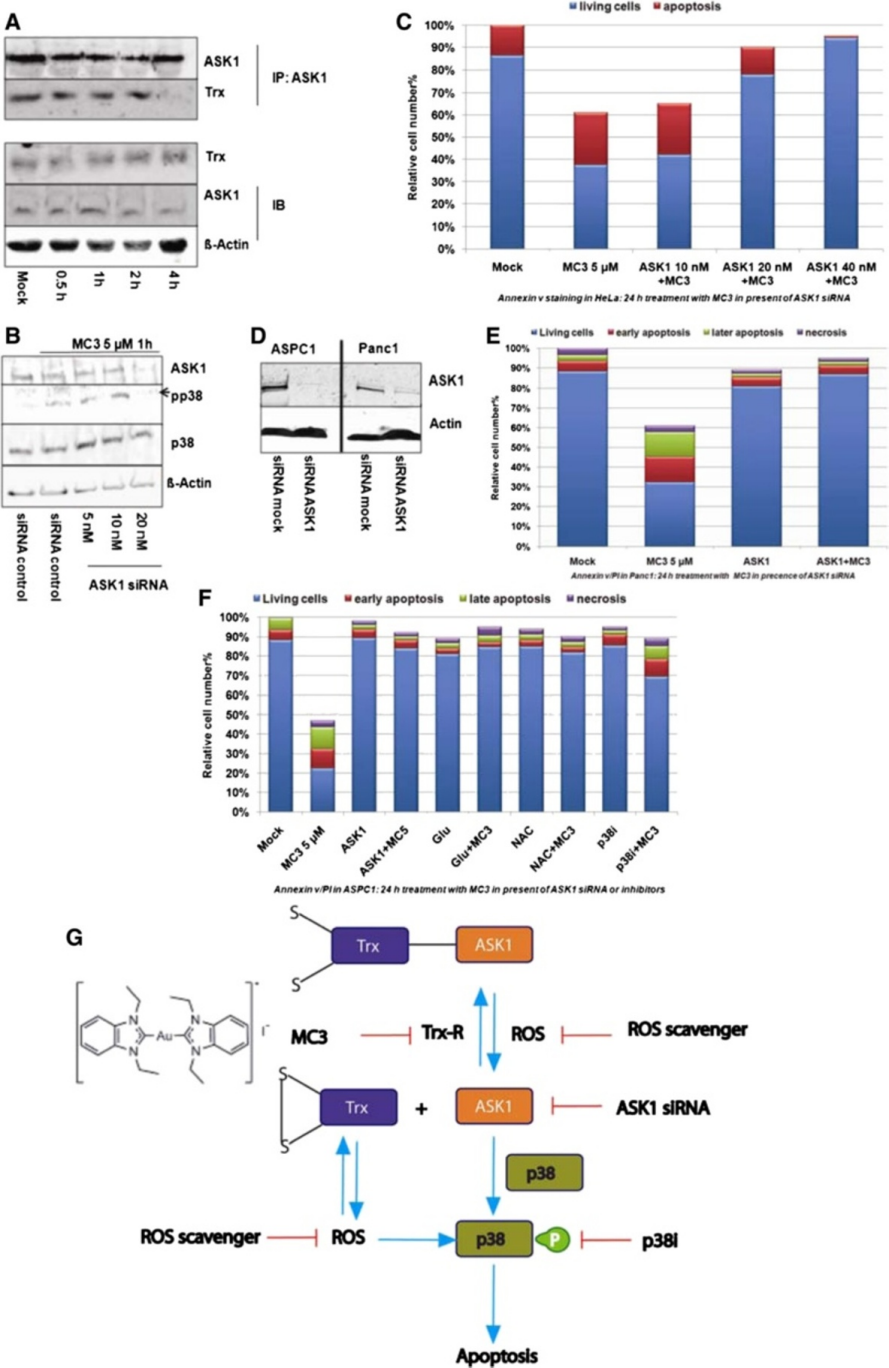
**The role of ASK1 in MC3-mediated cellular apoptosis. (A)** MC3 interrupted the binding between ASK1 and Trx. Panc1 cells were incubated with 5 μM MC3 as indicated. The cells were collected for immunoblot detected by Trx and ASK1 antibodies and for immunoprecipitation precipitated with ASK1 antibody and then immunoblotted with ASK1 and Trx antibodies. **(B)** ASK1 siRNA inhibited phophorylation of p38-MAPK. HeLa cells were transfected with ASK1 siRNAs for 48 h. The cells were treated with 10 μM MC3 for 1 h and collected for immunoblot. No target siRNA was used in the transfection as negative control. **(C)** Resistance to MC3 in ASK1-deficient HeLa cells. After 48 h transfection with ASK1 siRNAs HeLa cells were incubated with 5 μM MC3 for 24 h and the apoptotic cells were analyzed by FACS staining with annexin v/PI. **(D)** 50 nM of ASK1 siRNA was sufficient to knockdown endogenous ASK1 in ASPC1 (left) and Panc1 (right) after 48 h detected by immunoblotting. **(E)** ASK1 siRNA rescued MC3-induced cell apoptosis in annexin v/PI assay in Panc1. **(F)** ROS scavengers, p38i and ASK1 siRNA attenuated cellular damage upon MC3 in ASPC1 after 24 h treatment. **(G)** Schematic model of MC3 induced cell apoptosis in pancreas. *The densitometric analyses of corresponding immunoblots are provided in supporting information.*

Disruption of the interaction between ASK1 and Trx can activate ASK1-p38-MAPK signaling [22, 23]. To investigate whether activation of p38-MAPK was a result of releasing ASK1 from Trx caused by blocking the reductive recycling of Trx upon MC3, we used an ASK1 antibody to precipitate cellular ASK1 and examined the level of Trx binding to ASK1. After 4 h MC3 treatment the level of Trx in complex with ASK1 was clearly reduced, while ASK1 and Trx levels were not changed in the whole cell lysates (Figure 6A and Additional file 1: Figure S6), demonstrating that MC3 suppressed ASK1 binding to Trx.

To further analyse the role of ASK1 in MC3-induced apoptosis, we genetically reduced the level of ASK1 using siRNA in HeLa cells and detected lack of ASK1 resulting in the reduction of p38-MAPK activity without changing total p38 levels in response to 5 μM of MC3 (Figure 6B and Additional file 1: Figure S7). We used annexin v to label apoptotic cells after 24 h treatment with MC3 at 5 μM in HeLa cells transfected with increasing concentrations of ASK1 siRNA and observed a clear rescue with 20 nM of ASK1 siRNA (Figure 6C). We also transfected Panc1 with ASK1 siRNA (Figure 6D, right and Additional file 1: Figure S8) and found a similar reduction of MC3 toxicity (Figure 6E). However, a higher amount of siRNA (50 nM) was required to achieve this effect (Figure 6D and Additional file 1: Figure S8). Finally, we examined the influence of p38i, ROS scavengers and ASK1 siRNA on the activity of MC3 in ASPC1 cells. Comparably to the results in Panc1, viability of ASPC1 cells under MC3 treatment was markedly elevated in presence of one of the inhibitors, including ASK1 siRNA, ROS scavenger (Glu and NAC) and p38i (Figure 6F).

## Discussion

Pancreatic cancer, also known as pancreatic ductal adenocarcinoma, is one of the most incurable tumors with a rate less than 5% of 5-year survival [31]. Gemcitabine (Gem) is one of the FDA approved small molecules recommended to treat pancreatic cancer in advanced and metastatic stages with a modest, but statistically significant advantage in survival versus 5-fluorouracil [32]. Nevertheless, many pancreatic tumors like Panc1 are insensitive to Gem [46], also confirmed with our assay at concentrations of up to 20 μM. By contrast, MC3 showed an approximately 100-fold higher cytotoxicity in both pancreatic cancer cell lines and low toxicity against the non-cancer cell line HFF, suggesting a beneficial use of MC3 in gemcitabine resistant pancreatic cancer.

MC3 is a gold(I) NHC complex recently reported by our group and has been identified as a TrxR inhibitor [9, 10]. TrxR is required to convert oxidized Trx into its functional reductive form, which can scavenge ROS and consequently improves cell viability under oxidative stress [14]. Our results demonstrated that MC3 blocks this mechanism. As a results, the level of ROS was increased and mitochondrial membrane potential was decreased in Panc1 in response to MC3 treatment, whereas ROS scavengers reduced cell lethality (Figure 6G). Thus, the disruption in redox balance due to inhibition of TrxR should be the main mechanisms of MC3 mediated cell death, and explained by the following pathways.

Trx is a physiological inhibitor of ASK1 based on direct interaction and thus termination of ASK1-p38-MAPK-dependent apoptosis [22, 23]. Given that this interaction only occurs with Trx in its reduced form [24], MC3 logically reduced the binding of ASK1 to Trx as we showed in Figure 6A. As a result, free released ASK1 led to phoshorylation of p38-MAPK, which could be detected in all three cell lines investigated, including Panc1, ASPC1 and HeLa (wild type). In ASK1-deficient cells, p38-MAPK remained mostly in its inactive form, confirming that ASK1 is required for p38-MAPK activation upon MC3 treatment (Figure 6G).

p38-MAPK is a member of the mitogen-activated protein kinases family [47]. In comparison to other members, namely Erk1/2-MAPK and JNK-MAPK, which are often activated in human tumors and associated with cell proliferation, migration and metastasis, p38-MAPK had been described as a tumor suppressor, in particular under stress conditions [48]. The p38-MAPK pathway can be regulated by transcriptional and post-transcriptional mechanisms to influence cell death signaling and pro- and anti-apoptotic Bcl-2 proteins [47]. Cells with suppressed p38-MAPK signaling either using ASK1 siRNA or chemical p38 inhibitor were resistant to MC3 in Panc1, ASPC1 and HeLa cells, supporting the major role of the ASK1-p38-MAPK signaling axis in the induction of cell death by this gold(I) complex (Figure 6G). MC3-treated cells underwent G2/M arrest, showing constantly phosphorylated p53 and decreased Bcl-xL levels. Inhibition of p38 blocked phosphorylation of p53 and increased cell survival. Our results are in good agreement with previous findings, showing that p38 can regulate cell death through phosphorylation of p53, which leads to p53 stabilization and triggers cell cycle arrest in the G2/M phase [49].

## Conclusions

Taken together, the balance between intracellular ROS and redox capacity is crucial for maintaining cellular homeostasis and plays an imminent role in the capacity of cancer cells to cope with the oxidative stress associated with increased metabolic activity of rapidly growing cells found particularly in advanced stage tumors. Treatment with gold(I) NHC complexes interferes with this balance and leads to activation of the ASK1-p38-MAPK cascade and in consequence triggers apoptosis in gemcitabine-resistant pancreatic cancer cells. This very specific active profile of a gold(I) NHC complex indicates an interesting potential clinic benefit in chemotherapy of resistant cancers.

## Methods

### Materials

1,3-Diethylbenzimidazoliumiodide (MC1), chloro-(1,3-diethylbenzimidazol- 2-ylidene)gold(I) (MC2), [di-(1,3-diethylbenzylimidazol-2-ylidene)]gold(I) iodide (MC3), [triphenylphosphine-1,3-diethylbenzylimidazol-2-ylidene)]gold(I) iodide (MC4) were synthesized as described [9, 10]. Structures and purities were ascertained by ^13^C- and ^1^H-NMR, and mass spectroscopy and elemental analyses. N-acetyl-L-cysteine (NAC), glutathione (Glu), gemcitabine (Gem) and p38 inhibitor (p38i, SB203580) were purchased by Sigma-Aldrich (Germany). p38 (cat: #9212), pp38 (T180/Y182, cat: #9215), PARP (cat: #9542), pp53 (S15, cat: #9284) and pATF2 (T71, cat: #5112) were obtained from cell signaling (NEB, Germany). ASK1 (cat: sc-7931) and Trx (cat: sc-20146) were from Santa Cruz (Germany).

### Cell culture

HeLa, Miapaca, Panc1 and HFF cells were cultured in DMEM, and ASPC1 and BxPC3 in RPMI1640 containing 10% FBS and 1% Pen/Strp [32] under 5% CO_2_ at 37°C in a humidified atmosphere and treated with compounds as indicated in the text. For depletion of ASK1, siRNA oligonuleotides were designed and synthesized by Riboxx (Riboxx GmbH, Radebeul, Germany), based on the gene with number NM_005923.3 and used for transfection of cells. Sequences are: ASK1a: AUUUAGAUGAAAUACAGG (guide) and CCUGUAUUUCAUCUAAAU (passenger); ASK1b: AUAUUAUCUACUCGCUG (guide) and GCAGCGAGUAGAUAAUAU (passenger); ASK1c: AUUUAAAAUACUCACU (guide) and GAGUGAGUAUUUCUAAAU (passenger); ASK1d: UUCAAUGAUUGUACAG (guide) and GCUGUACAAUCUUGAA (passenger). The two most efficient sequences were ASK1a and ASK1d. Riboxx®FECT reagent (Riboxx, Radebeul, Germany) was used to get maximal transfectional efficiency according to the manufacturer’s instructions. After 48 h transfection in 10% FBS, cells were treated with compounds as indicated in the text.

### Sulforhodamine B assay (SRB assay) and (3-(4,5-Dimethylthiazol-2-yl)-2,5-diphenyltetrazolium bromide assay (MTT assay)

Effects on cell growth were determined using the SRB assay and MTT assay at an initial cell density of 5,000 cells/well in a 96-well plate as previously reported [9, 50].

### ROS formation assay

Panc1 cells were plated in 12-well plate at a density of 200,000 cells/well and cultivated in standard condition for 24 h before cells were treated with compounds as described in the text. Cells were collected by trypsinization and centrifugation with 200 g (1500 rpm), and resuspended in FACS buffer (1% BSA in PBS) containing 5 μM dihydroethidium (DHE, Sigma-Aldrich). After 15 min incubation at room temperature in the dark cells were washed with FACS buffer, and immediately analyzed using a FACSCalibur flow-cytometer (Becton Dickinson) and CellQuest Pro (BD) analysis software (FACS analysis). Excitation and emission settings were 488 nm and 564–606 nm (FL2 filter), respectively. One representative result is presented from at least three-independent experiments showing comparable results.

### Apoptosis assay

Panc1 and ASPC1 cells were seeded at a density of 200,000 cells/well in a 12-well plate under standard conditions and treated with chemicals as indicated in the text. After 24 h incubation cells were trypsinized and collected by centrifugation. The annexin v/PI staining assay (BD, Germany) was used to analyze cell apoptosis according to the manufacturer’s protocol. Briefly, the cells were re-suspended in the binding buffer and stained with FITC-conjugated annexin v for 15 min, followed by PI staining for 5 min at room temperature. The cell suspension was immediately analyzed by FACS. An aliquot of the re-suspended cells was used to determine the cell number after treatment, using a hemacytometer and trypan blue. The relative amount of cells was calculated as the number of trypan blue negative cells divided by the number in the mock treatment.

### Cell cycle analysis

Panc1 cells were seeded at a density of 200,000 cells/well in a 12-well plate under standard conditions. The cells were treated as designed, trypsinized and collected as mentioned above. The pellets were fixed in 70% Ethanol at least for 24 h, washed two times with ice-cold PBS and resuspended in 500 μL PBS. Cell suspensions were incubated with RNase A (50 μg/mL) for 30 min at 37°C and sequentially stained with PI (50 μg/mL) for 1 h and analyzed by FACS. At least two-independent experiments were performed.

### Detection of Bcl-xL by flow cytometry

Panc1 cells were cultivated under standard conditions (200,000 cells/well in a 12-well plate) and treated with various concentrations of MC3 as indicated for 24 h. Afterwards cells were trypsinized, collected, fixed in 4% paraformaldehyde for 10 min at 37°C and permeabilized in 90% methanol for 30 min on ice. Cells were blocked with 0.5% BSA solution in PBS, incubated with Alexa Flur ® 488-conjugated Bcl-xL (cat: #2767, cell signaling, NEB) for 1 h at RT, washed with PBS and analyzed by FACS. Excitation and emission settings were 488 nm and 564–606 nm (FL2 filter), respectively.

### Mitochondrial membrane potential

Panc1 cells (200,000 cells/well in a 12-well plate) were cultivated in DMEM with 10% FBS and treated with compounds as indicated. Cells were then stained with 2.5 μM JC-1 (5,5,6,6-tetrachloro-1,1,3,3- tetraethylbenzimidazolylcarbocyanineiodide, Sigma-Aldrich) for 30 min at 37°C, collected, and analyzed by FACS. Excitation and emission settings were 488 nm, 515–545 nm (FL1 channel) for JC monomers, and 564–606 nm (FL2 channel) for JC aggregates. Comparable results were obtained from at least in two-independent experiments.

### Immunoblot

Cell extracts were homogenized in urea-lysis buffer (1 mM EDTA, 0.5% Triton X-100, 5 mM NaF, 6 M Urea, 1 mM Na_3_VO_4_, 10 μg/mL Pepstatin, 100 μM PMSF and 3 μg/mL Aprotinin in PBS). Enhanced chemiluminescence (ECL) immunoblot analysis was performed. For this 40 μg of total protein was resolved on 10% SDS-PAGE gels and immunoblotted with specific antibodies. Primary antibodies were incubated at a 1:1,000 dilution in TBS (pH 7.5) with 0.1% Tween-20 and 5% BSA/nonfat milk with gentle agitation overnight at 4°C. The proper secondary antibodies were incubated in TBS (pH 7.5) with 5% BSA/nonfat milk and 0.1% Tween-20 at a 1:5,000 dilution for 1 h at room temperature. Comparable results were obtained from at least in two-independent experiments.

### Immunoprecipitation

Immunoprecipitation was performed according to the manufacture’s protocol (Cell Signaling, Frankfurt, Germany). Briefly, cells were lysed in lysis buffern (100 mM TRIS/HCl pH 7.5, 150 mM NaCl, 1 mM MgCl2, 0.25% NP-40, 5 mM NaF, 1 mM Na_3_VO_4_, 10 μg/mL Pepstatin, 100 μM PMSF and 3 μg/mL Aprotinin) and incubated with Rabbit serum (DAKO) for 1 h on ice and Agarose A/G-protein bead (Santa Cruz) for 30 min. Supernatant was immunoprecipitated with ASK1 (1:100, Santa Cruz) overnight at 4°C. Immune complexes were precipitated with Agarose A/G-protein bead for 2 h at 4°C min and analyzed by immunoblotting. Clean-Blot IP Detection Reagent (Thermo) was used to reduce background caused by denatured and blotted IgGs according to manufacture’s protocol. Comparable results were obtained in at least two-independent experiments.

### qRT-PCR

Quantitative reverse-transcription real-time-PCR was performed using a Light Cycler 480 (Roche, Germany) following a reference protocol from the manufacturer. Briefly, total RNA was isolated from cells using TRIzol (Qiagen, Germany). cDNA was generated by reverse-transcription using random primers and equivalent quantities of RNA. qPCR was performed using the SYBR Green PCR master mix (Roche, Germany) and the following primer pairs (MWG, Eurofins, Germany): ATF2 (5 s: CAGCGTTTTACCAACGAGGA; 3as: GAATCTTGTTGGTGTTGGGGTC); TP53 (5 s: CCTCACCATCATCACACTGGAAG; 3 s: CCTTTCTTGCGGAGATTCTCTTCC); Stat1 (5 s: GGAAAAGCAAGCGTAATCTTCAGG; 3as: GAATATTCCCCGACTGAGCC); and Actin (5 s: CTGACTACCTCATGAAGATCCTC; 3as: CATTGCCAATGGTGATGACCTG) as internal control. Results were obtained in three-independent experiments. Programs: preincubation, 1 cycle, 95°C, 5 min; quantification, 48 cycles, 95°C 10 sec, 60°C 10 sec, 72°C 20 sec; melting curve, 95°C 5 sec, 65°C 15 sec; cooling, 40°C.

### Wound healing assay

Panc1 cells were plated at high density (200,000 cells/well) into 12-well plates and grown to confluence. The scratch was made by a sterile P-200 micropipette in the middle of each well. Cells were then washed three times with PBS and treated with MC3. Photographs of the same fields were taken at the beginning, after one day and at the end of the experiments (2 days).

### Colony formation assay

Panc1 cells were plated into 12-well plate and grown to 70% confluence. Then cells were treated with MC3 for 24 h, harvested and re-plated into six-well plates with 2,000 cells/well. Medium were changed every 5 days. After 3 weeks, cells were stained with a staining solution containing 6% glutaraldehyde and 0.5% crystal violet for 1 h.

### Protein microarray analysis

Phosphorylated proteins were quantified using sandwich ELISA microarrays. The microarrays are based on the ArrayStrip™ platform (Alere Technologies GmbH, Jena, Germany). A detailed description of the assay protocol and information on reagents for this assay have been previously reported [41].

### Statistical analysis

Data were expressed as the mean ± SD of at least three independent experiments. Statistical significance of comparisons was assessed using the Student’s t-test (Microsoft Excel) and one-way ANOVA analysis. *P* < 0.05 was considered statistically significant.

## Electronic supplementary material

Additional file 1: Figure S1: Densitometric analysis of proteins in Figure 4A. **Figure S2.** Densitometric analysis of proteins in Figure 4B. **Figure S3.** Densitometric analysis of proteins in Figure 4F. **Figure S4.** Densitometric analysis of proteins in Figure 4G. **Figure S5.** Densitometric analysis of proteins in Figure 5C. **Figure S6.** Densitometric analysis of proteins in Figure 6A. **Figure S7.** Densitometric analysis of proteins in Figure 6B. **Figure S8.** Densitometric analysis of proteins in Figure 6D. (PDF 3 MB)

## Abbreviations

PDAC: Pancreatic ductal adenocarcinoma
NHC: *N*-heterocyclic carbine
TrxR: Thioredoxin Reductase
Trx: Thioredoxin
ROS: Reactive oxygen species
p38-MAPK: p38 mitogen-activated protein kinase
ASK1: Apoptosis signal-regulating kinase 1.
