# Supplementary figures and images for: A TrxR inhibiting gold(I) NHC complex induces apoptosis through ASK1-p38-MAPK signaling in pancreatic cancer cells
Source: Mol Cancer. 2014 Sep 25;13:221. doi: 10.1186/1476-4598-13-221 (PMC4190468; doi:10.1186/1476-4598-13-221)

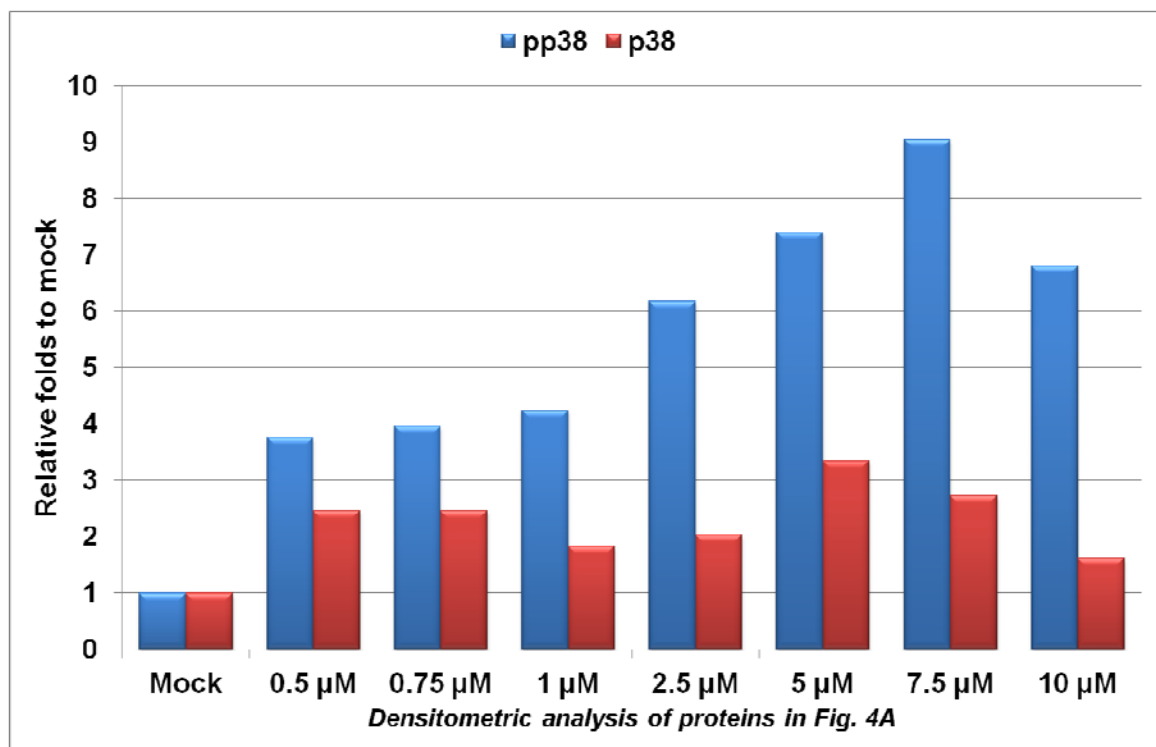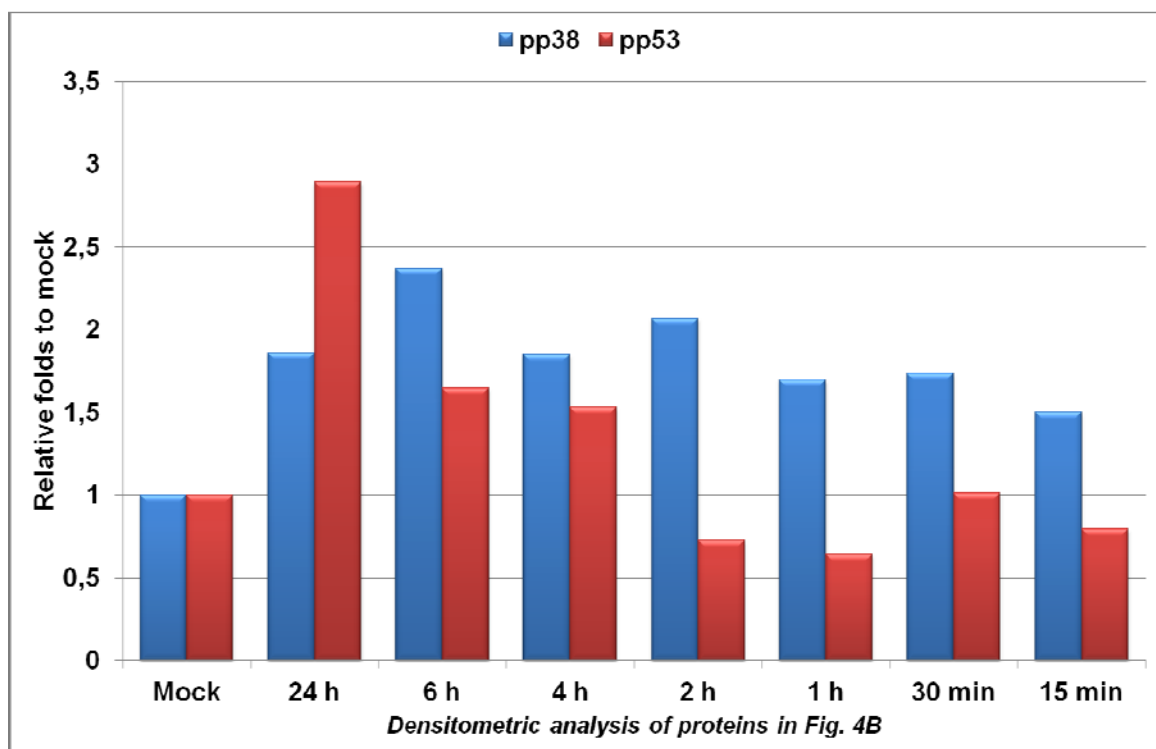

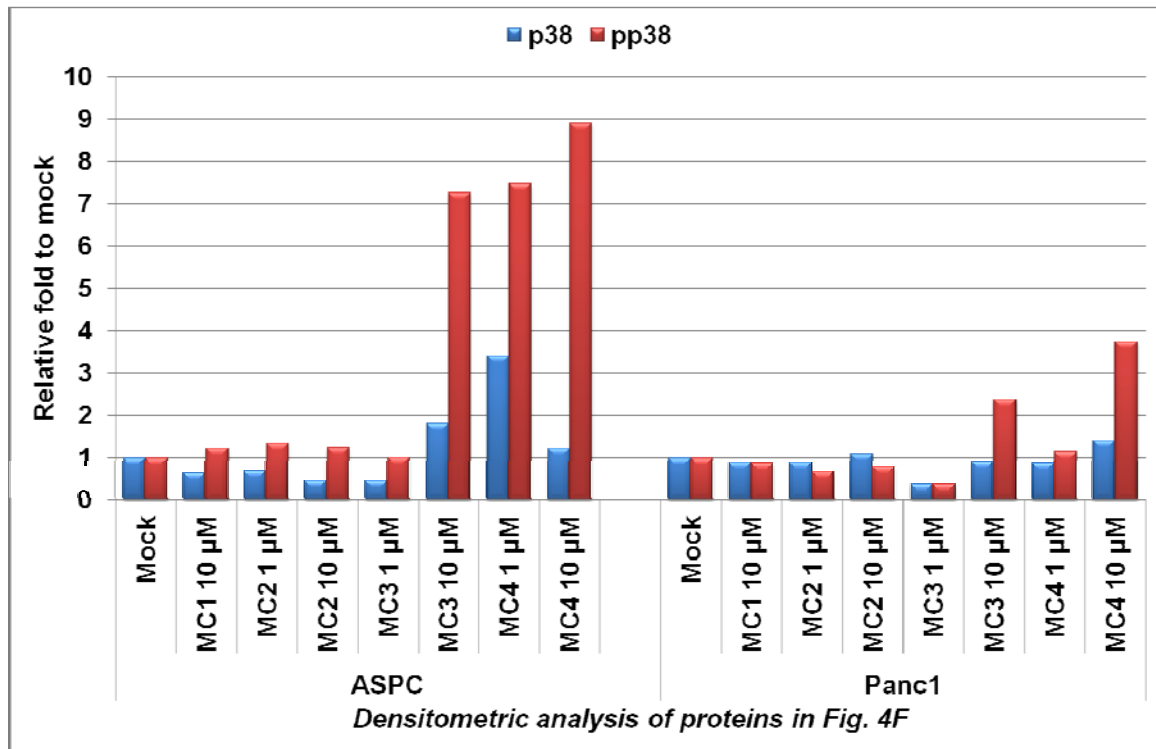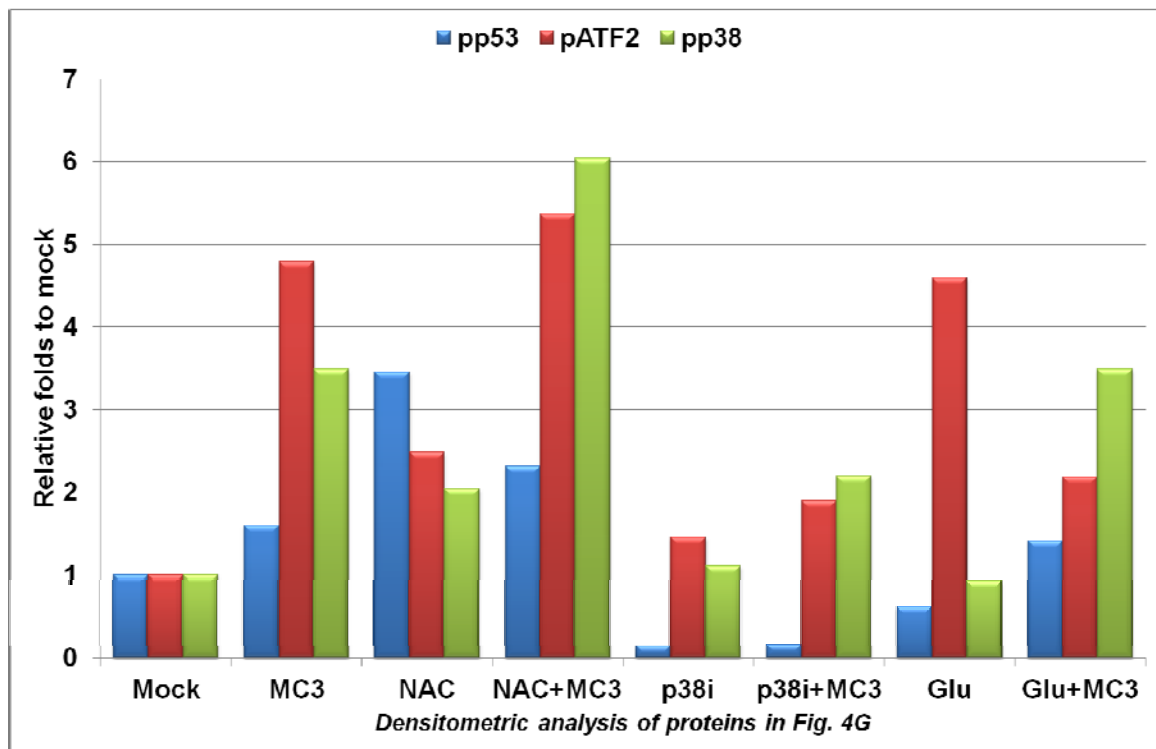

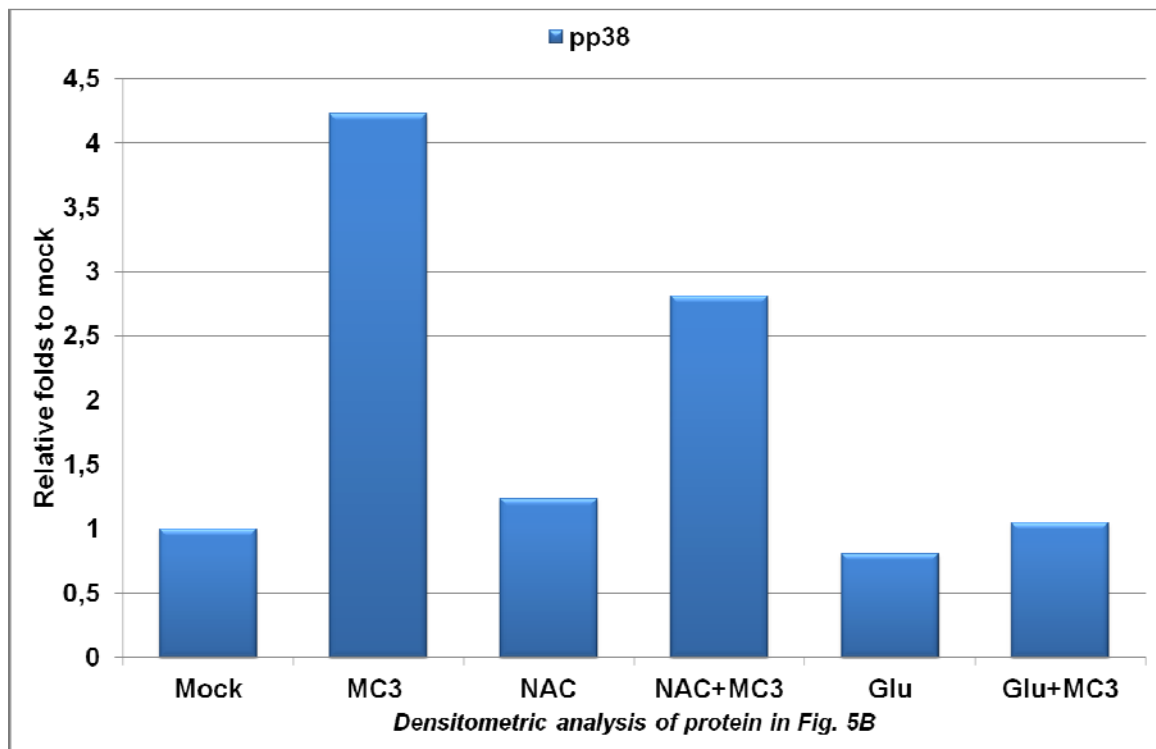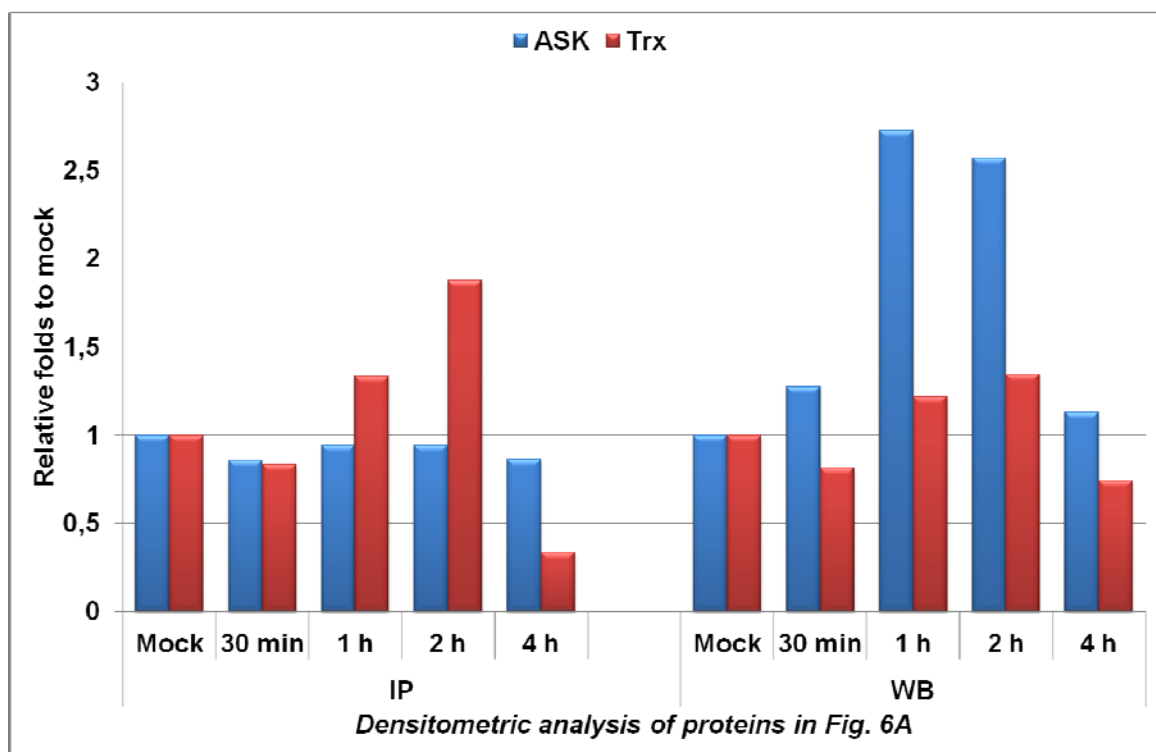

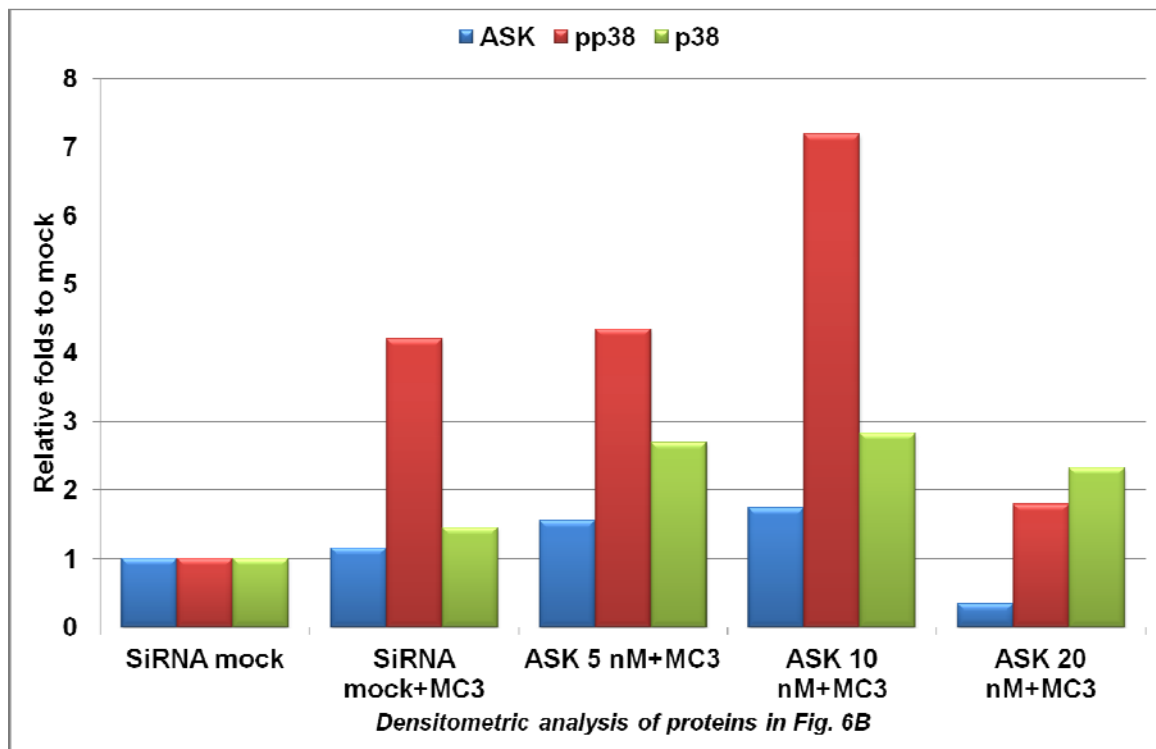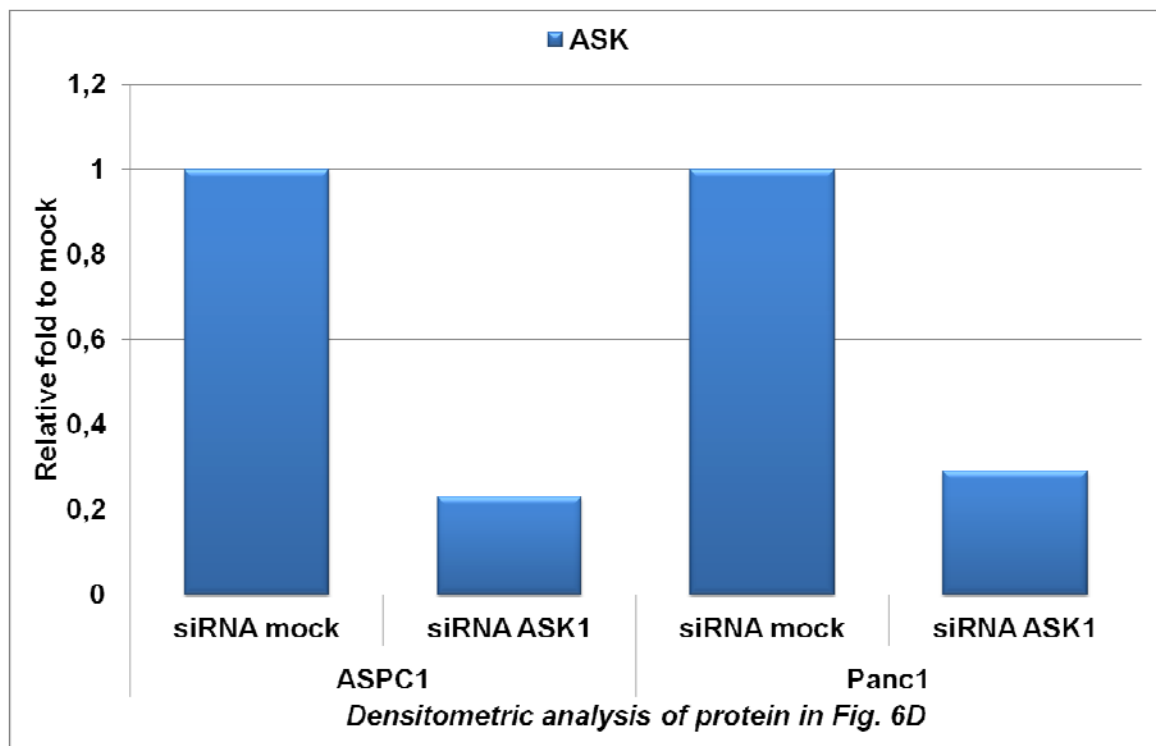

Supplement: Supplementary file 1 — Additional file 1: Figure S1: Densitometric analysis of proteins in Figure 4A. Figure S2. Densitometric analysis of proteins in Figure 4B. Figure S3. Densitometric analysis of proteins in Figure 4F. Figure S4. Densitometric analysis of proteins in Figure 4G. Figure S5. Densitometric analysis of proteins in Figure 5C. Figure S6. Densitometric analysis of proteins in Figure 6A. Figure S7. Densitometric analysis of proteins in Figure 6B. Figure S8. Densitometric analysis of proteins in Figure 6D. (PDF 3 MB) [file 12943_2014_1424_MOESM1_ESM.pdf]
